# Supplementary material for: Forward variable selection enables fast and accurate dynamic system identification with Karhunen-Loève decomposed Gaussian processes
Source: PLoS One. 2024 Sep 20;19(9):e0309661. doi: 10.1371/journal.pone.0309661 (PMC11414993; doi:10.1371/journal.pone.0309661)
Supplement: Attachment — Submitted filename: ResponseToReviewers2.docx [file pone.0309661.s005.docx]

Thanks to the editor and both reviewers for their re-consideration of the manuscript. Our responses to the points raised in review follow:

**Reviewer 2**

Comment

- in the abstract the complexity is stated, but it is not explained what N and P stand for (same page 3, line 18 and following. N=number of points, n=number of dimensions/features?)

Response

We have added definition of both N and P terms to both the abstract and the manuscript.

Comment

- equation (4): one could write in the sum i=1 ... P, then it is immediately clear that P is the number of terms in the expansion

Response

We have made this change.

Comment

- the style of Figure 1 (size, visability, axis ticks) could be improved

Response

We have increased the size of Figure 1.

**Reviewer 3**

Comment

The overall presentation of the work is good. Authors presented most of their funding in detail. However a small problem is on the definition of hyper parameters (HP), in some cases a form of optimization is applied to models and in some cases authors stated that “(Results were not particularly sensitive to the hyperparameter values”. It would be a good idea to present a detailed section or at least a table where the author provided full information on HP. HP is a crucial parameter that can significantly affect the overall performance of algorithms. Although authors mentioned that their models' HP are tuned, they did not provide a fully clear information on how they tuned it and whether the HP of counterpart methods are tuned optimally or not. Authors should add a section about this matter and novel approaches in this topic for the possible readers and clearly explain that if the hyper-parameters are not selected accurately or properly even the state-of-the-art methods would fail when compared with a tuned traditional model.

Response

We thank the reviewer for their insightful comment on the importance of hyperparameters (HPs) in model performance. We agree that HPs play a crucial role, which is why we have taken care to provide comprehensive information on HP selection in our manuscript. In our treatment of hyperparameters we have adhered to a number of principles:

1. Transparency and Reproducibility: All hyperparameters used in our experiments are explicitly listed in the text within the experimental section. This approach ensures full transparency and enables reproducibility of our results. We believe this level of detail in the main text is more appropriate than a separate table, given the diversity of methods compared and their distinct sets of hyperparameters.
2. Balancing Rigor with Practical Applicability: Our approach to HP selection balances rigorous comparison with real-world applicability. While we acknowledge the importance of HP optimization for achieving peak performance, we argue that comparisons using reasonable, near-default settings are equally valuable. This reflects common usage scenarios where extensive HP tuning may not be feasible due to time or resource constraints. Our goal is to provide insights that are relevant both for in-depth research and practical applications.
3. Strategic Approach to HP Optimization: Our decision to perform HP sweeps was strategically based on the initial performance differences observed between methods:

a) For methods showing significant performance gaps when using reasonable HP values (e.g., neural networks and decision trees vs. the KL decomposed GP), we deemed extensive HP optimization unnecessary. The substantial performance difference suggests that even with optimal HPs, these methods are unlikely to bridge the observed gap.

b) For methods demonstrating comparable performance (specifically SINDy), we conducted thorough HP sweeps to ensure a fair and robust comparison. This approach is detailed in the manuscript, reflecting our commitment to rigorous evaluation where it most impacts our conclusions.

4. Implications of Our Findings: The significant performance advantage of our KL decomposed GP over other methods, even with reasonable HP settings, suggests a fundamental strength of our approach. This difference likely stems from the inherent characteristics of the methods rather than suboptimal HP configurations.
5. Future Considerations: While we believe our current methodology provides a robust comparison, we acknowledge that exhaustive HP optimization for all methods could offer additional insights, particularly for closer comparisons or in domains where optimal performance is critical regardless of computational cost. This could be an avenue for future research.

In conclusion, our approach strikes a balance between methodological rigor and practical relevance. By clearly reporting all hyperparameters and our decision-making process for optimization, we ensure transparency and reproducibility while also reflecting realistic usage scenarios. We believe this methodology provides valuable insights for both researchers and practitioners in the field.

We added a discussion of these points to the revised manuscript.
